# Anti-synthetase syndrome presenting with interstitial lung disease

**DOI:** 10.31138/mjr.28.4.212

**Published:** 2017-12-22

**Authors:** Panagiotis Athanassiou, Markos Kostopoulos, Aikaterini Tzanavari, Anestis Spyridis, Ifigenia Kostoglou-Athanassiou

**Affiliations:** 1 Department of Rheumatology, St. Paul’s Hospital, Thessaloniki, Greece,; 2 Department of Endocrinology, Asclepeion Hospital, Athens, Greece

**Keywords:** anti-synthetase syndrome, mechanic’s hands, interstitial lung disease, myopathy, azathioprine

## Abstract

Anti-synthetase syndrome is an idiopathic inflammatory myopathy characterized by interstitial lung involvement, arthritis, Raynaud’s phenomenon, mechanic’s hands and fever. The case of anti-synthetase syndrome in a young female is reported. The patient presented with interstitial lung disease, initially subclinical muscle involvement, Raynaud’s phenomenon, arthritis and mechanic’s hands. Induction therapy was administrated, that consisted of intravenous methylprednisolone and cyclophosphamide, followed by azathioprine and prednisone with good response, and finally, complete remission. The presence of mechanic’s hands in a patient with lung involvement should prompt for specific investigations such as anti-synthetase antibodies and muscle enzymes. Anti-synthetase syndrome should be aggressively managed by immunosuppressive therapy, as interstitial lung disease is a severe manifestation of the disease which may affect quality of life and life expectancy.

## INTRODUCTION

Anti-synthetase syndrome is an autoimmune disorder characterized by myositis, interstitial lung disease, arthritis, fever, Raynaud’s phenomenon, thickened and roughened skin over lateral and palmar surface of fingers, termed as “mechanic’s hands”.^1^ The syndrome is characterized by the presence of anti-synthetase autoantibodies, namely anti-Jo-1 in cases of chest involvement or myositis as well as anti-threonyl-tRNA synthetase (anti-PL-7 and anti-PL-12) in cases of lung disease.^2^ Interstitial lung disease develops in most patients with anti-Jo-1 anti-synthetase syndrome. It often presents with sudden or gradual onset of shortness of breath on exertion, sometimes causing intractable dry cough, and may lead to pulmonary hypertension. Fever is present in about 20% of patients, and may occur at the onset of the disease or recur with relapses. Myositis is the most pertinent symptom characterizing the vast majority - >90% - of patients, and is associated with anti Jo-1 antibodies, proximal muscle weakness causing difficulty getting up from a chair or climbing chairs and pain in the muscles. Specific muscle group involvement may result in swallowing difficulties and aspiration pneumonia, while weakness of the respiratory muscles may lead to shortness of breath. Almost 50% of the patients experience joint pains or arthritis; most often symmetrical arthritis of small joints of hands and feet. The manifestation of mechanic’s hands involves thickened skin of tips and margins of fingers. Raynaud’s phenomenon is observed in about 40% of patients with some of them having myositis-specific nail fold capillary abnormalities. Myositis may be associated with environmental exposure to ultraviolet radiation, stressful life events and muscle overexertion, collagen implants, infectious agents such as retroviruses and bacteria, and certain drugs and chemicals. In the paper herein, we present a case of anti-synthetase syndrome presenting with interstitial lung disease, in which immunosuppressive treatment with pulse methylprednisolone and cyclophosphamide followed by azathioprine had a good response.

## CASE DESCRIPTION

**Figure 1.**
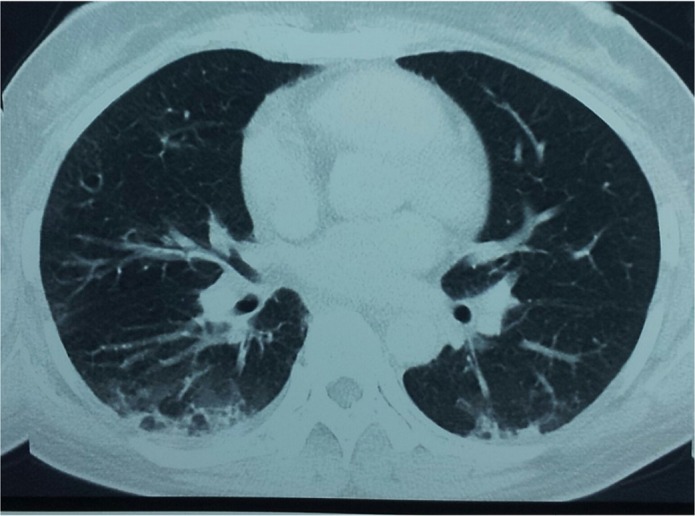
High resolution chest CT scan showing ground glass findings in the pulmonary bases bilaterally and thickening around bronchi.

**Figure 2.**
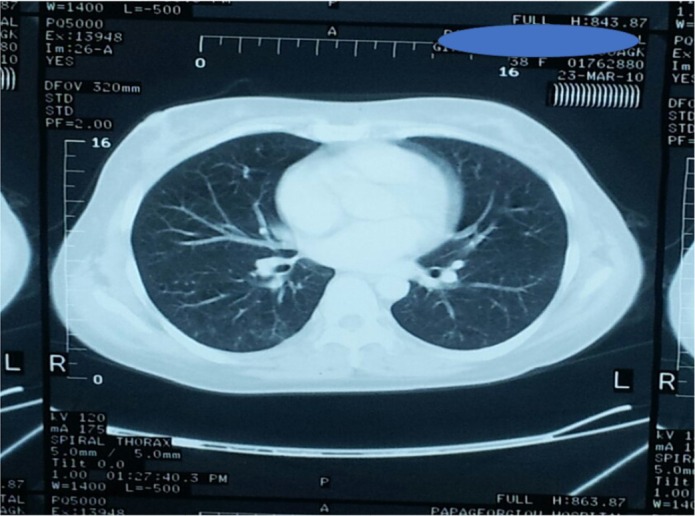
High resolution chest CT scan showing improvement of the lung disease.

A 37-year-old female patient presented to the emergency department with painful edema of upper extremities. Laboratory investigations revealed creatine kinase (CPK) at the region of 2,500 U/L (normal range 35–175 U/L). She did not report fatigue or muscle weakness. She was discharged, but she did not attend the follow-up appointment. Two months later, she developed a hand skin rash with hyperkeratosis, scaly and roughened skin over the fingertips. She was hospitalized and on admission she had mechanic’s hands, arthritis of the wrists and knees, Raynaud’s phenomenon, CPK 8,511 U/L, and normal muscle strength. An electromyogram was within normal limits, a muscle biopsy was negative for myositis and vasculitis, alanine aminotransferase (ALT) was 291 U/L (normal range 8–40 U/L), aspartate aminotransferase (AST) 230 U/L (normal range 8–45 U/L) and lactate dehydrogenase (LDH) was 679 U/L (normal range, 0–248 U/L) (**Table 1**). Immunology screening revealed positive antinuclear antibodies (ANA) with titer of 1/160 and nucleolar pattern, positive anti-Jo-1 and anti-Ro/SSA autoantibodies, while tests for anti-La/SSB, anti-scl70, anti-SRM antibodies and rheumatoid factor were negative. HLA class I typing was A2,A19,A31,B13,B48,Cw6. A high-resolution chest computed tomography (HRCT) scan showed ground glass opacities at the bases bilaterally (**Figure 1**) and thickening around bronchi. Spirometry revealed restrictive type pulmonary disease with forced vital capacity (FVC) at 77.9% predicted, (forced expiratory volume) FEV1 at 74.8% predicted and total lung capacity (TLCO) at 65.2% predicted (**Table 1**). Echocardiogram on admission was unremarkable. On the basis of clinical, biochemical and radiologic findings the diagnosis of anti-synthetase syndrome was made. Due to pulmonary involvement pulse methylprednisolone was administered intravenously in the dose of 1 g/day for 3 consecutive days followed by pulse cyclophosphamide 750 mg/m^2^ IV once monthly for 6 months. The patient’s condition improved. Hematocrit was 43.5%, white blood cell (WBC) count 4080 cells/mm,^3^ erythrocyte sedimentation rate (ESR) 8 mm/h, C-reactive protein (CRP) 0.2 mg/dL (normal range <0.5 mg/dL) and biochemical markers of muscular involvement as well as lung function tests were improved (**Table 1**). A new HRCT scan of the lungs revealed improvement compared to the one performed six months earlier (**Figure 2**). Following the completion of cyclophosphamide pulse therapy, azathioprine was initiated at 50 mg twice daily as maintenance therapy in combination with methylprednisolone 4 mg/d, calcium and cholecalciferol. Eighteen months after her initial presentation the patient was considerably improved on both clinical and biochemical grounds with hematocrit at 38.7%, CRP, ESR and biochemical markers of muscular involvement within normal range (**Table 1**). Echocardiogram showed normal dimensions of left and right ventricle, and a systolic pulmonary arterial pressure of 28 mmHg.

**Table 1.** Biochemical findings and lung function tests.

	**day 1**	**6 m**	**18 m**	**36 m**	**48 m**
**CRP (mg/dl)**	2.9	0.2	0.1	0.3	0.2
**ESR (mm/h)**	58	8	10	14	7
**CPK (U/L)**	8511	22	58	55	75
**ALT (U/L)**	291	11	15	12	14
**AST (U/L**	230	13	25	7	12
**LDH (U/L)**	679	175	163	160	170
**FVC %**	77.9	67.9	69.4		78.8
**FEV1 %**	74.8	73.2	75.2		80.5
**TLCO %**	65.2	60.6	63.7		75.5

Biochemical findings and lung function tests in the patient with anti-synthetase syndrome and interstitial lung disease over the course of the disease, at presentation (day 1), 6, 18, 36 and 48 months later (CRP – C-reactive protein-normal range <0.5 mg/dl, ESR – erythrocyte sedimentation rate, normal range 0–20 mm/h, CPK normal range 35–175 U/L, ALT normal range 8–40 U/L, AST normal range 8–45 U/L, LDH normal range 0–248 U/L, FVC – forced vital capacity, normal range >80%, FEV1 – forced expiratory volume–one second, normal range >80%, TLCO – transfer factor of the lung for carbon monoxide, normal range >80%).

CRP: C-reactive protein, ESR: erythrocyte sedimendation rate, CPK: creatine kinase, FVC: forced vital capacity, FEV: *forced expiratory volume, ALT:* alanine aminotransferase, AST: aspartate aminotransferase, LDH: lactate dehydrogenase

**Figure 3.**
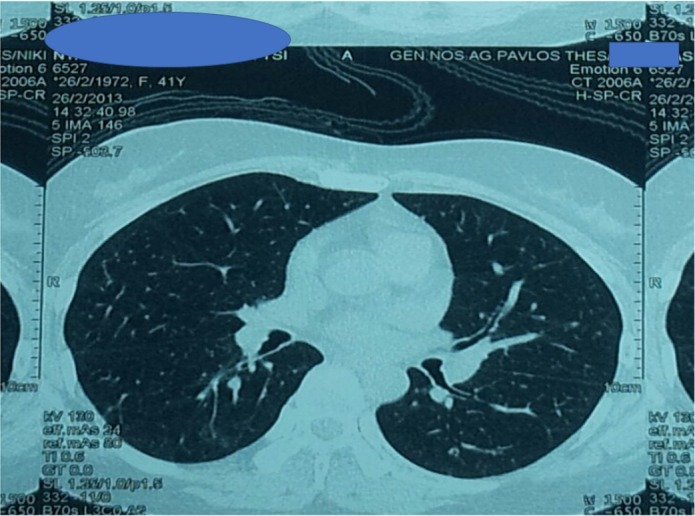
High resolution chest CT scan showing further improvement of the lung disease, in particular absence of ground glass opacities.

Three years after presentation, the patient was reevaluated and found to be stable. Laboratory investigations revealed normal CRP and ESR, normal muscle biochemistry while cardiac echography showed a pulmonary arterial pressure of 29 mmHg. HRCT showed improvement of the lung disease (**Figure 3**). The patient was on methylprednisolone 4 mg on alternative days, azathioprine 50 mg twice/d., calcium and cholecalciferol. Four years after initial presentation the patient was stable, a chest x-ray showed lack of active lesions in the lungs, and lung function tests were satisfactory (**Table 1**). Echocardiogram remained unremarkable. Hematocrit was 40.7%, ESR, CRP and biochemical markers of muscular involvement were within normal range (**Table 1**). Bone densitometry of left hip was compatible with mild osteopenia. Azathioprine was discontinued, while methylprednisolone 4 mg was continued on alternative days, and alendronate 70 mg/wk was added.

## DISCUSSION

Anti-synthetase syndrome is characterized by the presence of autoantibodies against one of many aminoacyl transfer RNA (tRNA) synthetases^3^ and it is a myopathy.^4^ During the 1980s aminoacyl tRNA synthetase autoantibodies were identified and linked to idiopathic inflammatory myopathies.^5^ During the 1990s, several groups recognized that patients with these antibodies had distinct clinical features.^6^ Individuals with anti-synthetase syndrome have a higher incidence of pulmonary involvement and symptoms characteristic of other connective tissue disorders, such as Raynaud’s phenomenon. In the present case lung involvement was a dominant finding along with Raynaud’s phenomenon. The hallmark of anti-synthetase syndrome is the presence of myositis-specific anti-synthetase antibodies.^3^ Of the anti-synthetase antibodies, anti-Jo-1, an anti-histidyl-tRNA synthetase, is most commonly identified. Less common anti-synthetase antibodies include anti-threonyl (anti-PL7), anti-alanyl (anti-PL12), anti-isoleucyl (anti-OJ), and anti-glycyl (anti-EJ) along with others infrequently tested in the clinical setting but reported in the literature. The patient reported herein had positive anti-Jo-1 antibodies. Anti-synthetase syndrome is a myopathy.^4^ The diagnosis of an inflammatory myopathy is based on clinical findings such as subacute development of symmetrical muscle weakness and signs such as laboratory investigations revealing skeletal muscle inflammation and muscle fiber degeneration and regeneration. The most easily available test to demonstrate skeletal muscle involvement is elevated levels of muscle enzymes, mainly CPK and others such as LDH, ALT, AST and aldolase. In the case described herein, CPK was very elevated on presentation, responded to treatment with immunosuppressive therapy and remained normal suggesting remission of the disease after four years of follow-up. It has been reported that anti-synthetase patients may not have classic myopathic symptoms, or myopathy may present in later stages of the disease.^7^ In accordance, it is noteworthy that on presentation our patient did not report any muscle weakness, despite extremely high levels of muscle enzymes denoting muscle involvement. Thus, in patients with interstitial lung disease findings in the hands such as scaling and hyperkeratosis should prompt anti-synthetase antibody testing and muscle enzyme measurement to rule out any subclinical myositis present.

In 2010, Connors et al.^8^ proposed diagnostic criteria for the anti-synthetase syndrome (**Table 2**), according to which the presence of an anti-aminoacyl tRNA synthetase antibody is required as well as one or more of several clinical features, namely, Raynaud’s phenomenon, arthritis, interstitial lung disease, fever and mechanic’s hands. In 2011, Solomon et al.^9^ also proposed diagnostic criteria for the anti-synthetase syndrome (**Table 3**), according to which the presence of an anti-aminoacyl tRNA synthetase antibody is required plus two major or one major and two minor criteria, the major criteria being interstitial lung disease and polymyositis or dermatomyositis, the minor criteria being arthritis, Raynaud’s phenomenon and mechanic’s hands. The patient described here fulfilled both the sets of criteria for the diagnosis of anti-synthetase syndrome proposed either by Connors et al.^8^ or Solomon et al.^9^ HRCT is used in the follow up of interstitial lung disease patients. In a 2015 study of patients with anti-synthetase syndrome^10^ traction bronchiectasis, ground glass opacities and reticulation were the most common findings at presentation. In the patient described above, ground glass opacities was the predominant finding at presentation.

**Table 2.** Proposed criteria for the anti-synthetase syndrome *Connors et al. 2010^8^*

**Required:** Presence of an anti-aminoacyl tRNA synthetase antibody
**plus** *one or more* of the following clinical features
Raynaud’s phenomenon
Arthritis
Interstitial lung disease
Fever (not attributable to another cause)
Mechanic’s hands (thickened and cracked skin on hands, particularly at fingertips)

**Table 3.** Proposed criteria for the anti-synthetase syndrome *Solomon et al. 2011^9^*

**Required:** Presence of anti-aminoacyl tRNA synthetase antibody	
**plus** *two major* or *one major and two minor criteria*:	
Major:	
1.	Interstitial Lung Disease (not attributable to another cause)
2.	Polymyositis or dermatomyositis by Bohan and Peter critieria
Minor:	
1.	Arthritis
2.	Raynaud’s phenomenon
3.	Mechanic’s hands

Immunosuppressive therapy is required for the treatment of anti-synthetase syndrome; the first line therapy being corticosteroids and azathioprine or mycophenolate mofetil being used as adjunctive therapy.^11^ Methotrexate has been used successfully for the treatment of anti-synthetase syndrome.^12^ Mycophenolate mofetil has also been used successfully in a case of refractory anti-synthetase syndrome.^13^ Rituximab has also been used successfully for the treatment of anti-synthetase syndrome,^14^ even in refractory cases.^15^ In the case described herein, corticosteroids as pulse methylprednisolone, followed by pulse cyclophosphamide and thereafter prednisone and azathioprine was used with a good response; full remission of the disorder being observed.

In conclusion, the case of a patient with anti-synthetase syndrome presenting with interstitial lung disease is described. The patient responded well to treatment with pulse methylprednisolone, thereafter pulse cyclophosphamide followed by prednisone and azathioprine with subsequent full remission of the disorder. Anti-synthetase syndrome is a myopathy in which interstitial lung disease may be observed. Patients with interstitial lung disease manifesting with thickened or roughened skin in the hands should be promptly evaluated for myopathy, so that the disorder is properly and promptly managed.
